# The Buerger’s rabbit model: a closer step to unravelling thromboangiitis obliterans?

**DOI:** 10.1186/s12959-024-00638-z

**Published:** 2024-07-29

**Authors:** Jia-Long Li, Kristine J. S. Kwan, Xue-Guang Lin, Jie Wang, Bo Chen, Yi-Jie Lu, Bo Wang, Shi-Shuai Xie, Jiong Zhou, Bo Yu, Ying Deng, Shuai Jiang, Jing-Dong Tang

**Affiliations:** Department of Vascular Surgery, Fudan University Pudong Medical Center, Shanghai Key Laboratory of Vascular Lesions Regulation and Remodeling, Shanghai, 201399 China

**Keywords:** Animal model, Arterial thrombosis, Thromboangiitis obliterans, Arterial occlusive disease, Vascular smooth muscle cell

## Abstract

**Objective:**

Thromboangiitis obliterans (TAO) remains clinical challenging due to its rarity and underwhelming management outcomes. This study aimed to describe a novel TAO rabbit model that demonstrates a closer resemblance to TAO.

**Methods:**

Thirty-six New Zealand rabbits underwent the surgical implantation of calibrated gelatin sponge particles (CGSPs) into their right femoral artery. The CGSPs were soaked in different solutions to simulate different types of thrombi: normal (NT; normal saline); inflammatory TAO thrombus (TAO; dimethylsulfoxide [DMSO]), and DMSO with methotrexate (MTX). All groups underwent clinical assessment, digital subtraction angiography (DSA) and histopathological analysis at time points day 0 (immediate), week 1 (acute), week 2 (subacute), and week 4 (chronic).

**Results:**

The TAO rabbit presented with signs of ischemia of the right digit at week 4. On DSA, the TAO rabbits exhibited formation of corkscrew collaterals starting week 1. On H&E staining, gradual CGSP degradation was observed along with increased red blood cell aggregation and inflammatory cells migration in week 1. On week 2, disorganization of the tunica media layer and vascular smooth muscle cell (VSMC) proliferation was observed. In the TAO rabbit, migrated VSMCs, inflammatory cells, and extracellular matrix with collagen-like substances gradually occluded the lumen. On week 4, the arterial lumen of the TAO rabbit was filled with relatively-organized VSMC and endothelial cell clusters with less inflammatory cells. Neorevascularization was found in the MTX-treated group.

**Conclusion:**

The novel TAO rabbit model shows a closer resemblance to human TAO clinically, radiographically, and histopathologically. Histological analysis of the IT progression in the TAO model suggests that it is of VSMC origin.

**Supplementary Information:**

The online version contains supplementary material available at 10.1186/s12959-024-00638-z.

## Introduction

Thromboangiitis obliterans (TAO) is a rare peripheral arterial disease (PAD) characterized by non-atherosclerotic, segmentally occlusive, and progressively inflammatory changes affecting the small and medium-sized vessels [1]. The prevalence of TAO in all patients with PAD varies across countries with different socioeconomic status, ranging as low as 0.5%—5.6% in Western Europe, but a much higher incidence among Ashkenazi Jews and natives of Indian, Korean, and Japanese ancestry [2]. Regardless, the decline in smoking and adherence to a more stringent diagnostic criteria has resulted a decrease in TAO prevalence over the past years [3]. Therefore, unlike atherosclerotic PADs, the attention for TAO has gradually diminished, especially when management often results with a poor prognosis [4].

Animal models are essential for understanding the pathophysiology and novel treatment alternatives for human diseases [5]. In vivo limb ischemia models of different chronicity have been established, including sheep, pig, rabbit, and rodents [6]. While large animals demonstrate anatomical and physiological similarities to humans, they are susceptible to limitations regarding accommodation, finance, and perioperative management, which can be overcame with small animals. Majority of designs were purposed for atherosclerosis research. Yet, the pathophysiology of TAO distinct itself from atherosclerotic diseases [7].

Rats naturally resist atherosclerotic development [8]. By injecting sodium laurate into the femoral artery of a rat, the drug supposedly induces endothelial cell (EC) damage that may lead to the aggregation of platelets in peripheral vasculature [9]. The sodium laurate rat (SLR) model is currently widely accepted as the TAO animal model and adopted in several pharmacological studies [10]. However, the onset of symptoms of lower limb ischemia is rather aggressive as the gangrened paw can fall off within 2 weeks. Although ultrastructural changes of SLR femoral arteries were comparable to TAO patients, there is no description regarding the histopathology of inflammatory thrombus (IT) that is also characteristic of TAO [11, 12]. Without a deeper understanding towards the pathophysiological process of TAO and incorrect application of animal research, blind attempts are likely made in finding a possible cure.

To provide a better experimental basis and reference for clinical management of TAO, we established a TAO rabbit model to observe the morphological, histopathological, and radiological changes of lower extremity artery. Results were further compared to human TAO and histopathological analysis was performed at different phases of IT progression.

## Materials and methods

### Experimental animals

Thirty-six New Zealand rabbits of male gender, aged 36 weeks, and weighing 2.0 – 2.5 kg, were provided by JIAGANSHENGWU (Shanghai, China). The rabbits were randomly divided into a control group with normal thrombus (NT; normal saline-soaked calibrated gelatin sponge particles [CGSP; 1 – 1.4 mm diameter; Alikon Co. Ltd., Hangzhou, China]), a IT group (CGSP soaked in dimethylsulfoxide [DMSO]), and a methotrexate (MTX)-treated IT group (MTX dissolved in DMSO at 10 g/L), with 12 animals in each group. All animals were treated and cared for in accordance with the National Institutes of Health [NIH] Guide for the Care and Use of Laboratory Animals (Revised, 1996), and all protocols were approved by the Fudan University Pudong Medical Center Institutional Animal Care and Use Committee.

### Animal model establishment

**Fig. 1:**
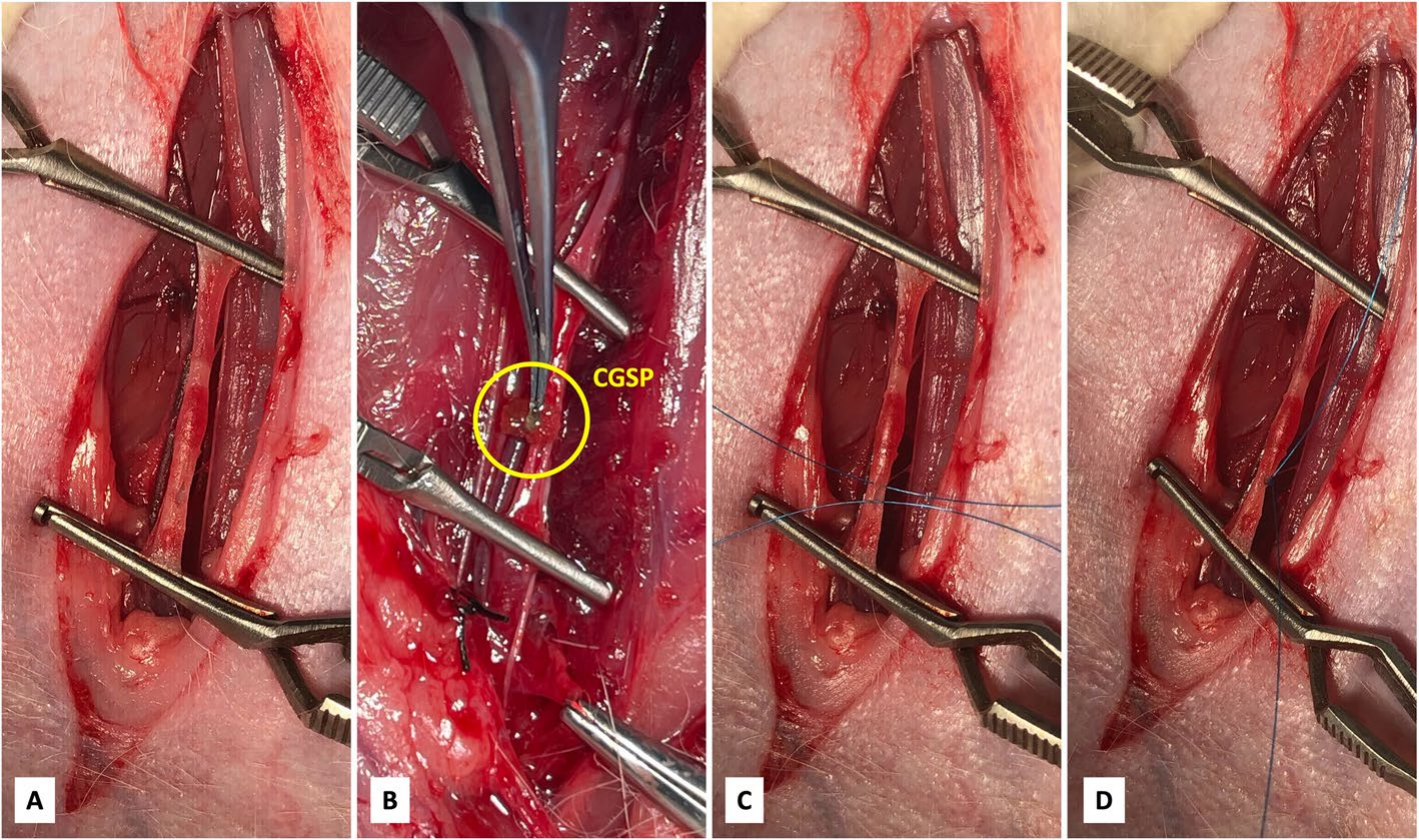
Schematic diagram of calibrated gelatin sponge particles (yellow circle) placement into the right femoral artery of the rabbit

General anesthesia was performed by slow injection of zolazepam hydrochloride (Zoletil™ 50#, Shanghai, China; induction dosage 0.8 – 1.0 ml, sustained for 0.2 – 0.3 ml) through the ear marginal vein. After successful anesthesia, the rabbit was placed on the operating table in the supine position. The right hind leg was shaved and sterilized with 95% alcohol. A longitudinal skin incision was made in the medial thigh, extending from the inguinal ligament to the knee, to expose the superficial femoral artery (SFA) (Fig. 1A). The SFA was dissected along its total length, freed, and controlled with vascular clamps at its proximal and distal ends. A 1 cm vertical incision was made to the near proximal segment of the SFA. Ten grains of CGSP (1000 – 1400 µm) were introduced to the incision opening with forceps (Fig. 1B). The SFA was closed with a 6–0 Prolene® (Ethicon, USA) (Fig. 1C, D). The distal vascular clamp was released prior freeing the proximal vascular clamp. The wound was closed when the distal SFA remained pale and no blood leakage was observed. After disinfection of the incision site, all animals, except for rabbits sacrificed on day 0, received ampicillin sodium (100 mg/kg/day; Bioss Antibodies, Beijing, China) intramuscularly for 3 days, beginning on the day of surgery.

### Digital subtraction angiography

DSA examinations were performed at week(s) 1, 2, and 4 time points postoperatively in all rabbits (*n* = 3 from each group). All procedures were carried out in the Shanghai Key Laboratory of Vascular Lesions Regulation and Remodeling with the Philips Azurion 3 M15 system (Philips Medical Systems, Netherland). Rabbits were anesthetized as mentioned above. Iodinated contrast material was dilated with water at a 3:1 ratio before manually injected into the abdominal aorta with an 18-gauge needle to visualize the circulation of the bilateral lower extremities. Blood flow of the experimented right lower limb was compared to the normal left limb.

### SFA harvest

Upon completing DSA, the rabbits were sacrificed by air embolization. The right SFA was exposed as mentioned above and the occluded site, as seen on DSA, was harvested. The intervened segment was carefully excised and cut into segments that were subsequently used for histological analysis.

### Histological analysis

Three independent tissue segments from each SFA (*n* = 3 per group and each time point) were removed and fixed in 4% formaldehyde solution. Preparations were according to Servicebio® (Wuhan, China) instructions. After dewaxing and hydration, the tissues were paraffin-embedded (JB-P5, Wuhan JunJie Electronics Co., Ltd., Wuhan, China) and sectioned (RM2016, Shanghai Leica Instrument Co., Ltd., Shanghai, China). HE standing (H&E Staining Kit G1003, Servicebio®) was then performed as follows: the tissues were first stained with hematoxylin dye solution for 3 – 5 min, rinsed, differentiated, washed, blued, and rinsed; the slices were dehydrated and eosin dye was added for 5 min; the slices were dehydrated and sealed with the neutral gum; and the slices were placed under light microscope (Nikon Eclipse E100, Nikon, Tokyo, Japan).

### Immunohistochemistry

Cellular constituents within the intimal and medial layers were semiquantified using immunohistochemical techniques with ICAM-1 and VCAM-1immunostaining. Sections (5 µm) of paraffin wax-embedded tissues were deparaffinized and heated in 20 × Tris–EDTA (pH 9.0) buffer (G1203, Servicebio®) in the microwave on medium heat for 10 min then low heat for 7 min. After natural cooling, the slide was placed in PBS (pH 7.4) and washed 3 times. Then, the slices were placed in 3% hydrogen peroxide solution, incubated at room temperature away from light for 25 min. After 3 washes with PBS, the sections were incubated with a matching blocking serum (10% normal goat or rabbit serum; G1209, Servicebio®) diluted in PBS and then incubated with ICAM-1 (GB11106; 1:800; Servicebio®), a marker for vascular inflammation, and VCAM-1 (GB113376; 1:500; Servicebio®), a marker for EC activation, overnight in a wet box at 4 °C. After 3 washes with PBS, the sections were incubated with goat anti-rabbit IgG (H + L) secondary antibody (1:200 dilution; GB23303; Servicebio®) for 50 min at room temperature. After 3 final washes with PBS and dried, DAB staining was prepared using the DAB chromogenic reagent for histochemical kit (Servicebio®) according to manufacturer’s instructions.

Obtained images of high power fields were digitized and analyzed using ImageJ (NIH, Bethesda, MD, USA). The areas of expression of ICAM-1 and VCAM-1 per total area was calculated by determining the area of brown pixels divided by the total area, multiplied by 100%.

### Data analysis

Statistical analysis was performed with SPSS 29.0 (IBM Corp., Armonk, NY, USA). To compare the variations across groups, the analysis of variance (ANOVA) with with post-hoc Tukey HSD test was used. The threshold of statistical significance was a 2-sided *P* value of < 0.05.

## Results

### Clinical presentation

**Fig. 2:**
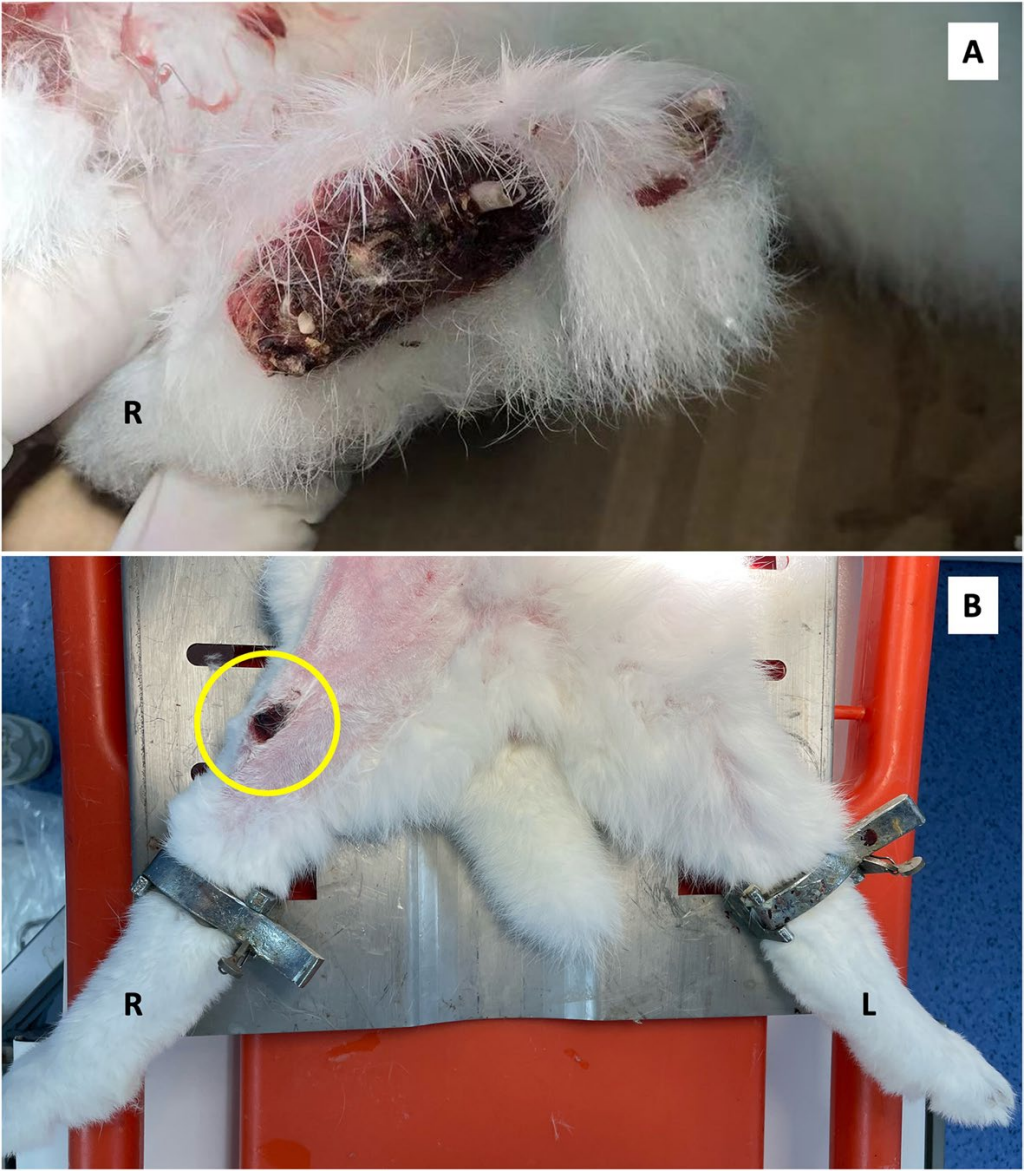
Ulceration of the lower limb digits (**A**) in the TAO rabbit model at week 4 and one presenting with ulceration above the anterior knee (**B**)

No rabbits died of surgical complications or spontaneously throughout the observation period. From day 0 to week 2, there was no obvious sign of lower limb ischemia. Mobility of rabbits was observed during feeding but no obvious difficulty in movement was observed. On week 4, all TAO rabbits showed signs of digital ulceration and dry gangrene of variable sizes (Fig. 2A) with one exception above the anterior knee (Fig. 2B).

### DSA images

**Fig. 3:**
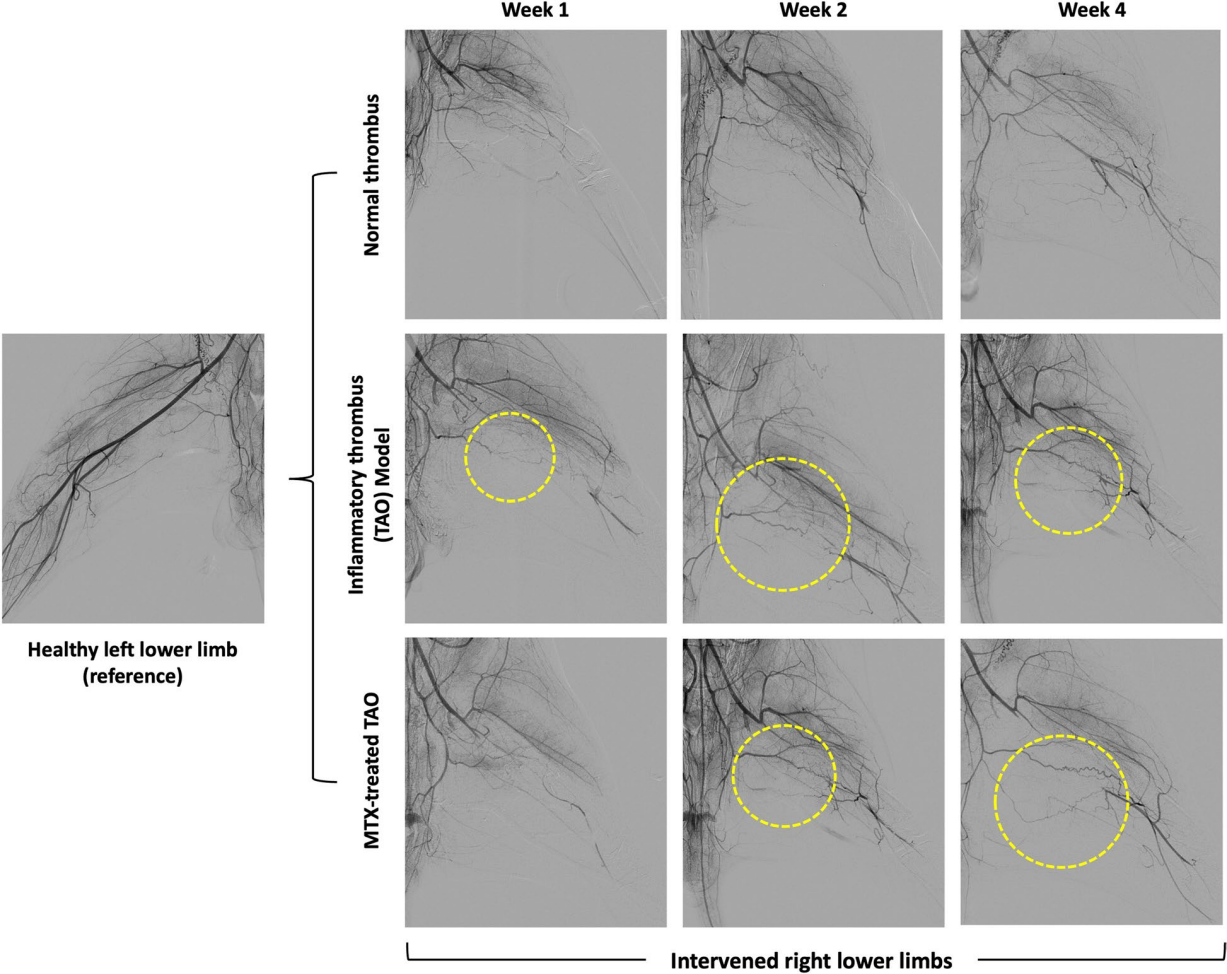
Digital subtraction angiography results showing occlusion of the right femoral artery. Corkscrew collateral formation can be seen in the inflammatory thrombus and methotrexate-treated groups (yellow circle). Restoration of distal blood flow can be seen beginning week 2

All images were summarized in Fig. 3. On week 1, all groups showed the absence of distal femoral arterial flow from the site of occlusion. In the TAO model, signs of corkscrew collateral formation were seen on week 1.

On week 2, all groups demonstrated the formation of collaterals. The TAO and MTX-treated TAO models showing obvious spiral-like corkscrew collaterals when compared to the NT group. Distal femoral arterial flow was gradually restored.

On week 4, the NT model showed thin streaks of femoral arterial flow in the CGSP-occluded segment, suggesting recanalization. The presence of corkscrew collaterals remains prominent in the TAO and MTX-treated TAO models that facilitated restoration of distal femoral arterial flow.

### Histopathological analysis of thrombus progression

**Fig. 4:**
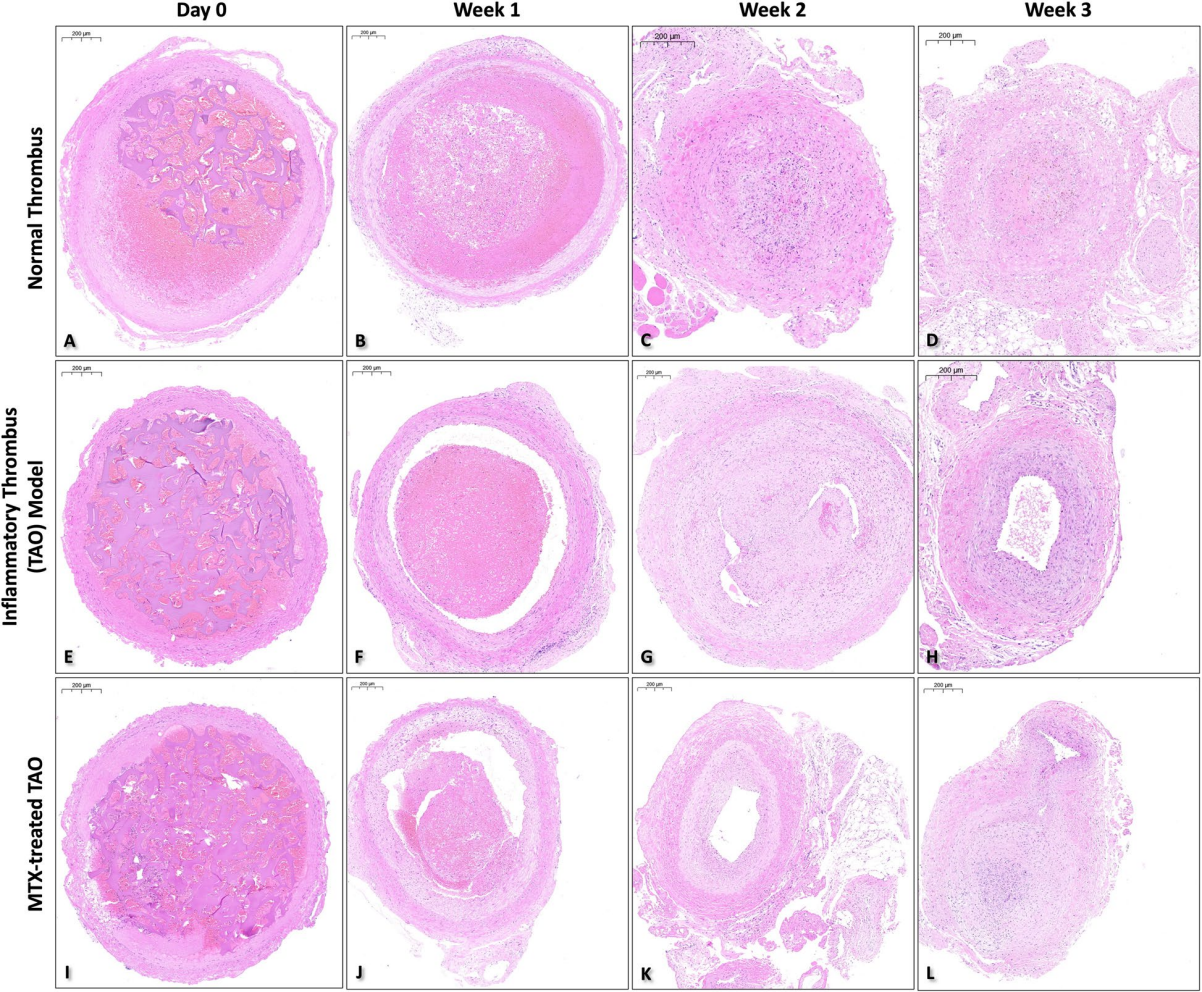
H&E Staining images (× 20 magnification; 200 μm) of different groups at different time points

Right femoral arteries from one rabbit of each group were harvested after CGSP insertion for reference (day 0). Intact and normal arterial morphology, CGSP within the lumen with red blood cells (RBCs) within the gaps was seen (Figs. 4A, E, I).

On week 1 acute thrombus phase, CGSP thickness have decreased with increased RBC aggregation and larger number of inflammatory cells present (Figs. 4B, F, J). In the NT group, the arterial morphology remained relatively normal. In the TAO and MTX-treated groups, folding of the internal elastic lamina (IEL) was seen (Fig. 4F, J).

On week 2 subacute thrombus phase, very little to no CGSP was found in all groups (Fig. 4C, G, K). Disorganization and proliferation of vascular smooth muscle cells (VSMCs), highly cellular thrombi with VSMC-like cells and inflammatory cells, and neorevascularization were seen in the NT and TAO groups at the media. In the TAO group, partial disruption of the endothelial layer and cellular infiltration, including VSMC-like cells and inflammatory cells, to the subintimal layer was seen. Duplication and disorganization of the IEL was prominent at the left upper quadrant of Fig. 4G. In the MTX-treated group, intimal hyperplasia was found (Fig. 4K).

On week 4 chronic thrombus phase, observations in the NT group remained similar to week 2 (Fig. 4D). In the TAO group, a complete second IEL layer was formed with VSMC-like cells and endothelial cells (ECs) reorganizing into a second media-like layer within the lumen (Fig. 4H). Less inflammatory cells were identified. In the MTX-treated group, intimal hyperplasia with neorevascularization was seen (Fig. 4L).

Throughout the progression, the vessel walls of all groups remained relatively intact. In order to identify the presence of VSMCs in the arterial lumen of the TAO group, random sections at weeks 2 and 4 were selected for α-SMA staining (Supplementary Fig. 1). Results suggest that cellular components of the IT included VSMCs.

### ICAM-1 and VCAM-1 staining

**Fig. 5:**
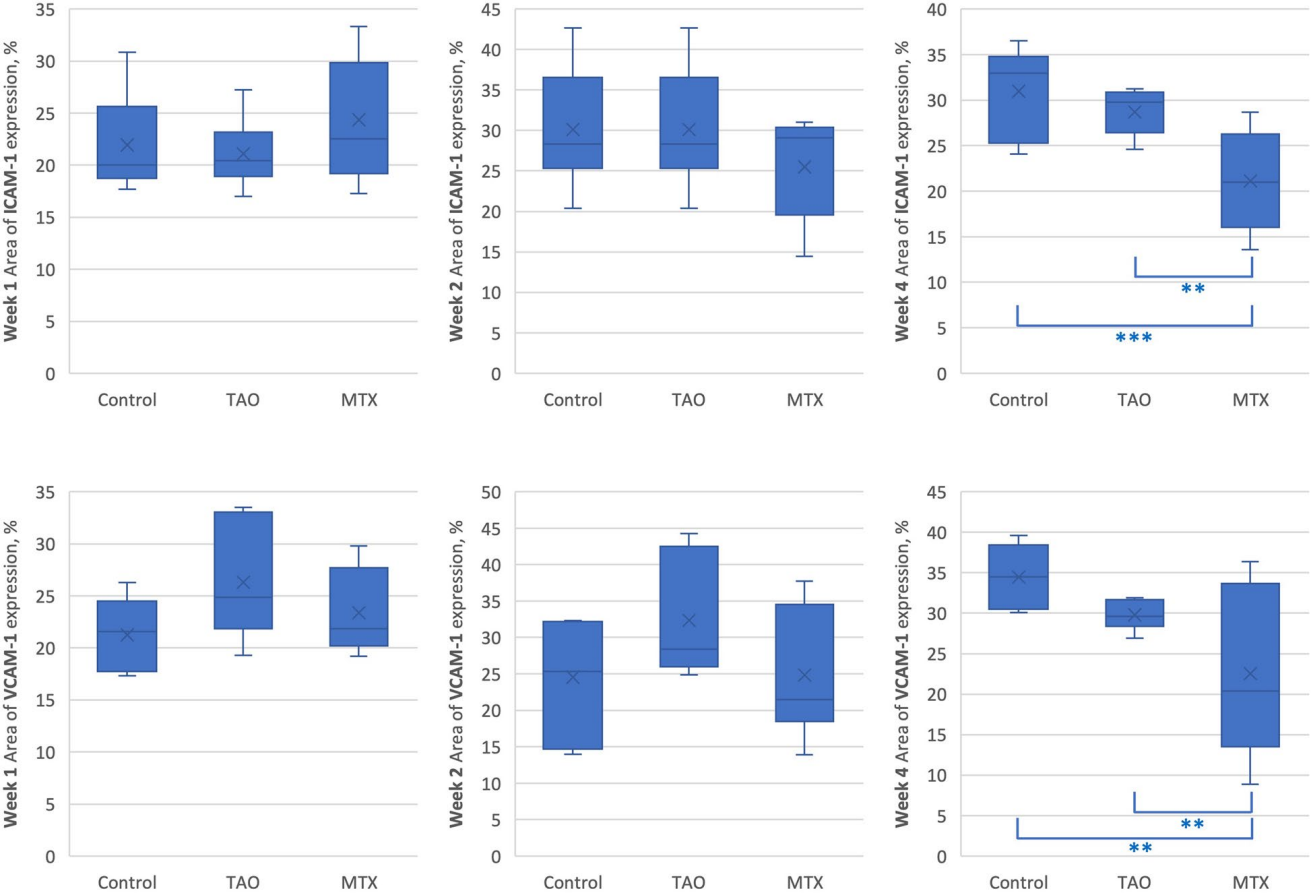
Summary and comparison of area of ICAM-1 and VCAM-1 expression between groups at a specific time point. *p* ≤ 0.05 *, *p* ≤ 0.01 **, *p* ≤ 0.001 ***

Figure 5 and Table 1 summarizes the means of ICAM-1 and VCAM-1 expression between groups at a specific timepoint. At timepoints week 1 and 2, the means of ICAM-1 and VCAM-1 between different treatment groups did not differ significantly. At timepoint week 4, the means of ICAM-1 between different treatment groups differ significantly, *F*(2,24) = 11.99, *p* < 0.001, η^2^ = 0.50 and the means of VCAM-1 between groups differ significantly, *F*(2,24) = 8.02, *p* = 0.002, η^2^ = 0.40.

**Table 1:** Comparison of area of ICAM-1 and VCAM-1 expression between groups at a specific time point

	Week	Area of expression, %						ANOVA *p* value	Post hoc Tukey’s test
		Control		TAO		MTX			
		Mean	SD	Mean	SD	Mean	SD		
ICAM-1	1	21.9	4.5	21.1	3.2	24.4	5.6	.302	-
	2	21.9	9.0	30.1	7.3	25.5	6.1	.286	-
	4	31.0	4.8	28.7	2.5	21.1	5.6	< .001	TAO, Control > MTX
VCAM-1	1	21.2	3.6	26.3	5.5	23.4	4.0	.073	-
	2	24.5	8.0	32.4	8.2	24.8	10.2	.098	-
	4	34.5	3.7	29.7	1.8	22.6	7.8	.002	TAO, Control > MTX

*TAO* Thromboangiitis obliterans, *MTX* Methotrexate, *SD* Standard deviation

^*^P < 0.05 considered statistically significant

**Fig. 6:**
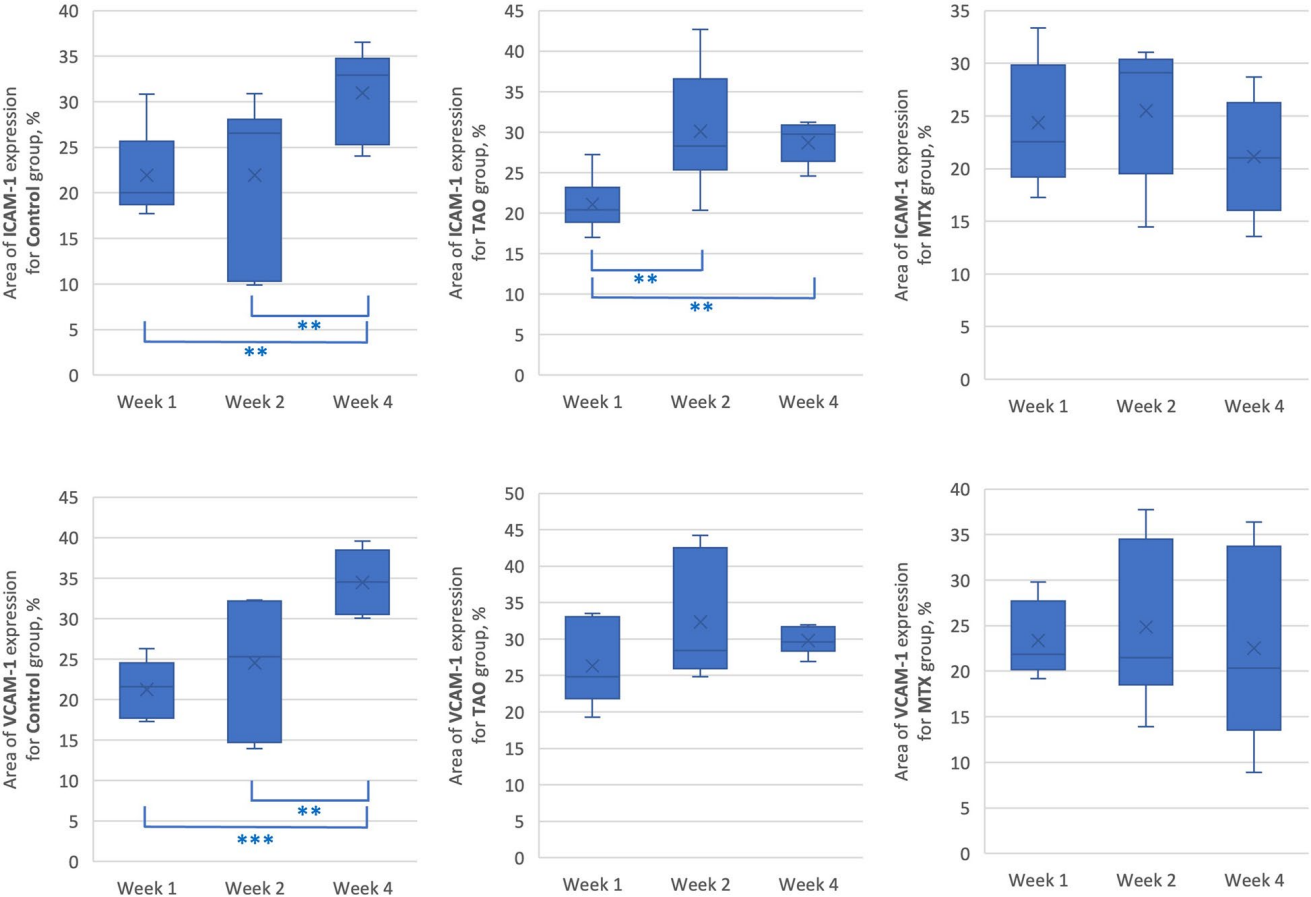
Summary and comparison of area of ICAM-1 and VCAM-1 at different timepoints for a specific group. *p* ≤ 0.05 *, *p* ≤ 0.01 **, *p* ≤ 0.001 ***

Figure 6 and Table 2 summarizes the ICAM-1 and VCAM-1 expression of each specific group with means at different time points. For the control group, the means of ICAM-1 between timepoints week 1, 2, and 4 differ significantly, *F*(2,24) = 5.98, *p* = 0.008, η^2^ = 0.33 and the means of VCAM-1 between timepoints week 1, 2, and 4 differ significantly, *F*(2,24) = 13.91, *p* < 0.001, η^2^ = 0.54.For the TAO group, the means of ICAM-1 between timepoints week 1, 2, and 4 differ significantly, *F*(2,24) = 9.09, *p* = 0.001, η^2^ = 0.43. However, the means of VCAM-1 did not show any significant difference between each timepoints. In the MTX-treated group, the means of ICAM-1 and VCAM-1 between different timepoints did not show significant differences.

**Table 2:** Comparison of area of ICAM-1 and VCAM-1 at different timepoints for a specific group

	Group	Area of expression, %						ANOVA* p* value	Post hoc Tukey’s test
		Week 1		Week 2		Week 4			
		Mean	SD	Mean	SD	Mean	SD		
ICAM-1	Control	21.9	4.5	21.9	9.0	31.0	4.8	.008	Week 4 > Week 1, Week 2
	TAO	21.1	3.2	30.1	7.3	28.7	2.5	.001	Week 4 > Week 2 > Week 1
	MTX	24.4	5.6	25.5	6.1	21.1	5.6	.271	-
VCAM-1	Control	21.2	3.6	24.5	8.0	34.5	3.7	< .001	Week 4 > Week 2 > Week 1
	TAO	26.3	5.5	32.4	8.2	29.7	1.8	.105	-
	MTX	23.4	4.0	24.8	10.2	22.6	7.8	.827	-

*TAO* Thromboangiitis obliterans, *MTX* Methotrexate, *SD* Standard deviation

^*^P < 0.05 considered statistically significant

## Discussion

This novel TAO rabbit is similar to the sodium laurate rat (SLR) model in terms of requiring surgical manipulation of a healthy femoral artery to induce peripheral limb ischemia and using cytotoxic substances to induce EC damage [3]. However, the TAO rabbit demonstrates a longer duration for the IT to evolve and allows for better understanding of the potential pathophysiological process of TAO. The onset of symptoms of lower limb ischemia was later and least aggressive than the SLR where the gangrened paw could fall off within 2 weeks. Additionally, we found the formation of corkscrew collaterals in DSA examination. Although it belongs to one of the diagnostic criteria of TAO, it is not a pathognomonic finding, yet still demonstrates a similarity to human TAO [5, 6].

Fibrin, platelets, RBCs, leukocytes, and neutrophil extracellular traps are the principal components of a typical thrombi [7]. Over time, the fibrin network begins to stiffen that eventually impairs responsiveness to antiplatelet therapy and thrombolysis [8]. TAO is characterized by its IT that has three different pathophysiological phases [9]. Histopathological observations made in the TAO group at different time points demonstrated a very similar phenomenon to what was previously described, which begins as a highly cellular thrombus with diminishing presence of RBCs and inflammatory cells, and increased organization of the of these cells with increased chronicity. Throughout this process, the vessel walls remained relatively intact.

In this TAO model, the highly cellular structure was highly suggested to be of VSMC origin. Under normal physiological conditions, VSMCs in the medial layer have high contractility and are spindle-shaped [10]. Under certain circumstances that induce EC injury, VSMCs may respond by expressing decreased levels of contractile proteins and increased levels of synthetic proteins that turn them into epithelioid-shaped dedifferentiated synthetic VSMCs [11]. The disorganization and proliferation of VSMCs in the medial layer and signs of migration towards the lumen with increased extracellular matrix was found in week 2 [12]. The possible formation of a new medial layer bordered within the new IEL layer was especially interesting. This abnormal growth may explain ‘lotus-root’ appearances witnessed in intravascular ultrasounds of arteries of TAO patients [13].

Should the IT be VSMC-EC clusters, then this would justify the limited to no response of thrombolytics even during the acute phase [14, 15]. When medical therapy fails, surgical intervention was preferred. However, previous experiences have documented the challenges in endovascular recanalization and prevention of restenosis even for immediate acute lower limb ischemia in TAO [16]. We believe that injury to the IT would trigger an aberrant VSMC-EC interaction and without the barrier effect of IEL during migration, such modes of interaction could possibly be accelerated in terms of direct contact [17]. Hence resulting in short-term restenosis or recurrent thrombosis as adherence, proliferation, and migration of VSMC can take over a week [18]. The significantly increased ICAM-1 and VCAM-1 levels in the TAO group starting week 2 may suggest that VSMC activity is at its peak. Whereas the gradual increase of ICAM-1 and VCAM-1 in the NT group may be due to inflammation from chronic thrombus [19]. MTX is used for the treatment of vasculitis, but its benefits for TAO, which is considered as an autoimmune vasculitis, is unknown [20, 21]. Since MTX is DMSO-soluble, it was designed to serve as a treatment group for the TAO model. The lowered ICAM-1 and VCAM-1 levels suggest a possible inhibition or therapeutic effect. However, in vivo studies demonstrated that DMSO combined with methotrexate exhibited synergistic cytotoxicity [22]. Therefore, without a comparison regarding the concentration of agents, it is difficult to determine the actual effect of MTX.

Several limitations need be addressed. Firstly, this experiment artificially induces arterial thromboembolism by implanting CGSP into a healthy peripheral artery segment. However, thrombosis of the peripheral arteries is mainly secondary to atherosclerosis and rarely occurs spontaneously [23]. The application of DMSO, with its reported effects of increasing RBC non-immune hemolysis, decreasing platelet aggregation, and inhibiting VSMCs migration and proliferation in vitro, paradoxically triggered VSMC cell-like migration and proliferation that eventually led to complete local arterial occlusion [24, 25]. The cytotoxic effects of DMSO is dose-dependent, where a low dose can present with antioxidant properties [26]. Recently, it has been mentioned that oxidative stress plays an important role in the pathogenesis of TAO [27]. It is well-established that cigarette smoking initiates oxidative stress, VSMC proliferation and death [28, 29]. However, further studies are necessitated to elucidate exact etiology of TAO. DMSO-soluble smoking particles have been used to study the association between tobacco and cardiovascular diseases, which may be utilized in future experimental designs to determine the exact role of smoking and its pathophysiological mechanism in TAO [30].

Secondly, results from the TAO rabbit model were from its preliminary experimental phase and would have especially benefited from larger samples for analysis. Statistical results from the small sample size can only demonstrate a possible trend but cannot provide any significant impact. The addition of adjuvant examinations, such as electron transmission microscopy, cytochemistry, staining (e.g.. Elastica van Gieson method), and other assays to aid in the identification of structures, understanding of cellular interactions, and design for potential therapeutic approaches. Unfortunately, this study faced restricted funds. Although the DSA finding of corkscrew collaterals and similar histopathological progression of IT suggested a closer resemblance to TAO, most of the phenomenon found in this study were purely descriptive and would require greater efforts for final validation.

Thirdly, a meaningful censoring is yet determined for the TAO model. TAO is a recurring progressive inflammatory process and patients are often admitted at a more advanced stage of the disease [31]. Unlike plaque buildup that causes stenosis in atherosclerotic disease that can be established by ligation of the artery, the pathological mechanism and progression of TAO is possible intraluminal from circulating autoreactive antibodies, which is why intervention was conducted from within the lumen [32, 33]. Rabbits have a higher thrombin clearance than humans despite a similar coagulation profile. DSA results also suggest it reasonable to consider week 1 as the acute phase of the TAO model and week 4 or beyond as a chronic phase [34, 35]. The period is comparatively longer than the SLR and could allow for better therapeutic research at different phases.

Nonetheless, observations from this study may likely contribute to future research, especially in the area of VSMC and its contribution to a variety of vascular diseases. TAO displays its own complex interplay between thrombosis and inflammation. The novel TAO rabbit model with IT is easily reproducible, and warrants for larger replications, extensive studies, advanced examinations to fully elucidate the observations made that may provide insight to the pathophysiology and management of TAO.

## Conclusion

This novel TAO rabbit model demonstrated a closer resemblance to human TAO clinically, angiographically, and histopathologically. The evolution of the IT suggested that it belonged to VSMC origin, which renders current management plans for TAO ineffective. Results were mainly descriptive. Further replications of this model and research may help elucidate the pathophysiology of TAO, as well as provide insight to its diagnosis and treatment.

### Supplementary Information

Supplementary Material 1: Supplementary Figure 1. α-SMA-stained section of superficial femoral artery of thromboangiitis obliterans rabbit model at (A) Week 2 and (B) Week 4. 10x magnification view showing the positive area of α-SMA suggesting VSMC involvement in inflammatory thrombus within the arterial lumen.

## Data Availability

No datasets were generated or analysed during the current study.
