# Supplementary figures and images for: The Buerger’s rabbit model: a closer step to unravelling thromboangiitis obliterans?
Source: Thromb J. 2024 Jul 29;22:69. doi: 10.1186/s12959-024-00638-z (PMC11285203; doi:10.1186/s12959-024-00638-z)

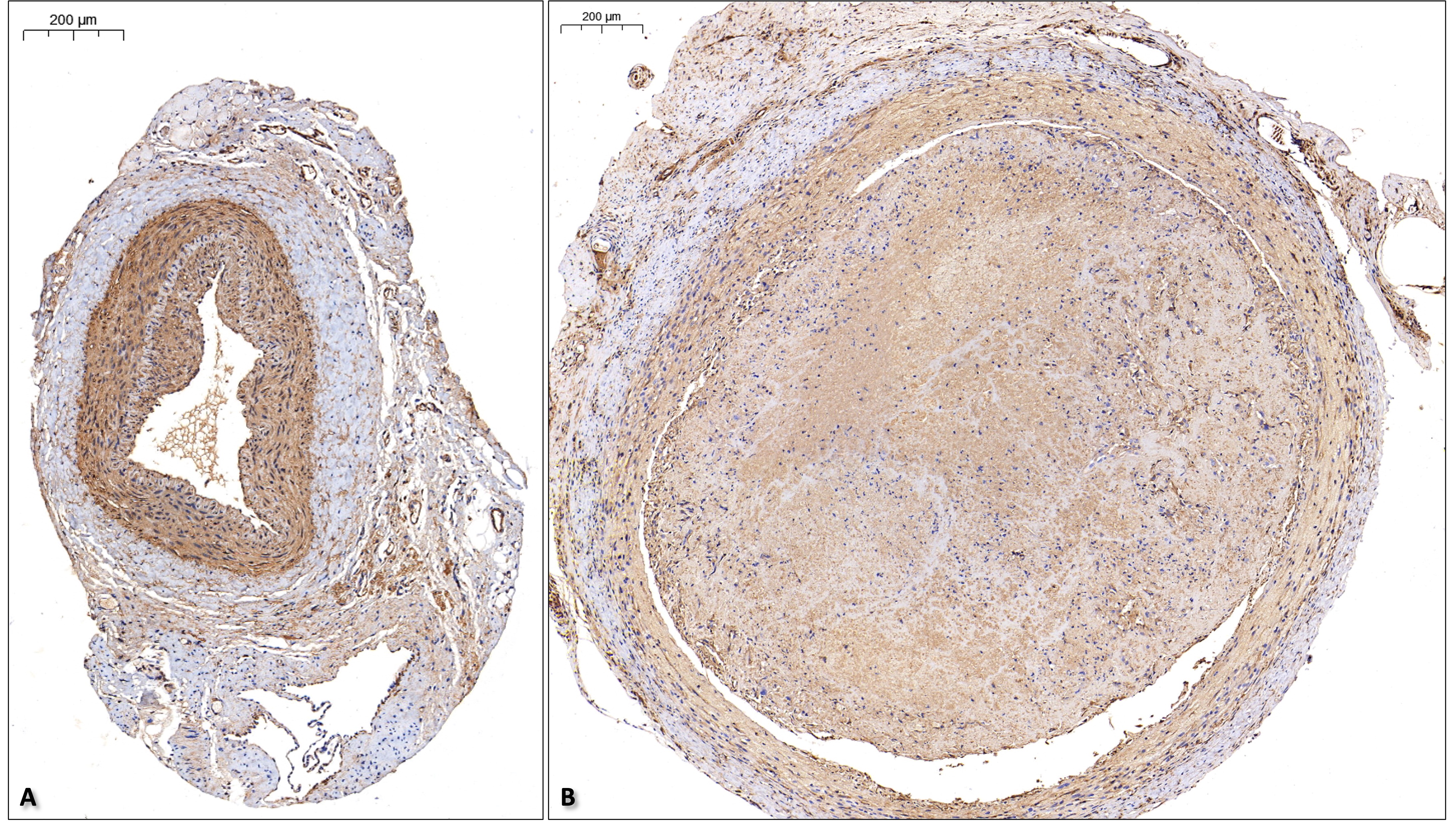

Supplement: Supplementary file 1 — Supplementary Material 1: Supplementary Figure 1. α-SMA-stained section of superficial femoral artery of thromboangiitis obliterans rabbit model at (A) Week 2 and (B) Week 4. 10x magnification view showing the positive area of α-SMA suggesting VSMC involvement in inflammatory thrombus within the arterial lumen. [file 12959_2024_638_MOESM1_ESM.jpg]
